# Recent Advances in the Anti-Inflammatory Activity of Plant-Derived Alkaloid Rhynchophylline in Neurological and Cardiovascular Diseases

**DOI:** 10.3390/pharmaceutics13081170

**Published:** 2021-07-29

**Authors:** Rajeswari Gopal Geetha, Surya Ramachandran

**Affiliations:** Cardiovascular Diseases and Diabetes Biology, Rajiv Gandhi Centre for Biotechnology, Thycaud P.O., Thiruvananthapuram 695014, India; rajeswarigopal@rgcb.res.in

**Keywords:** rhynchophylline, inflammation, atherosclerosis, neuroprotective, diabetes

## Abstract

Rhynchophylline (Rhy) is a plant-derived indole alkaloid isolated from Uncaria species. Both the plant and the alkaloid possess numerous protective properties such as anti-inflammatory, neuroprotective, anti-hypertensive, anti-rhythmic, and sedative effects. Several studies support the significance of the anti-inflammatory activity of the plant as an underlying mechanism for most of the pharmacological activities of the alkaloid. Rhy is effective in protecting both the central nervous system and cardiovascular system. Cerebro-cardiovascular disease primarily occurs due to changes in lifestyle habits. Many previous studies have highlighted the significance of Rhy in modulating calcium channels and potassium channels, thereby protecting the brain from neurodegenerative diseases and related effects. Rhy also has anticoagulation and anti-platelet aggregation activity. Although Rhy has displayed its role in protecting the cardiovascular system, very little is explored about its intervention in early atherosclerosis. Extensive studies are required to understand the cardioprotective effects of Rhye. This review summarized and discussed the various pharmacological effects of Rhy in neuro- and cardioprotection and in particular the relevance of Rhy in preventing early atherosclerosis using Rhy-loaded nanoparticles.

## 1. Introduction

**Figure 1:**
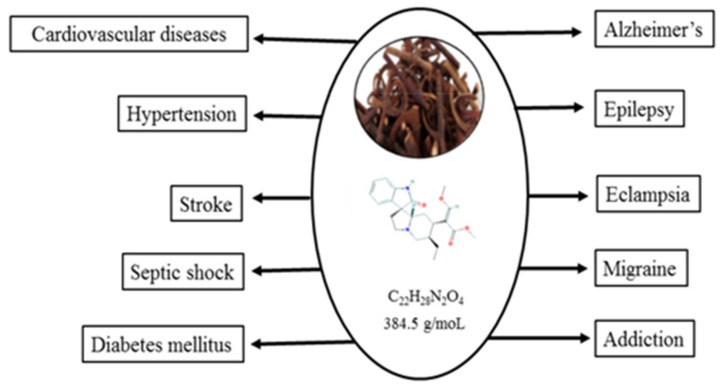
Biological effects of rhynchophylline.

Rhynchophylline (Rhy) is a plant-derived tetracyclic oxindole alkaloid found in certain species of Uncaria (Gouteng in Chinese, belonging to the family Rubiaceae). It is isolated from *Uncaria rhynchophylla* (Miq.) Jacks [1] *U. tomentosa* [2] and in leaves of *Mitrangya speciose* [3,4]. Using the hooks in the plant, Uncaria has been used in Traditional Chinese Medicine (TCM) as well as Japanese Kampo medicine for the treatment of infantile convulsion, cardiovascular disorders, headache, dizziness, hypertension, stroke [5,6], eclampsia, epilepsy, and other cerebral diseases [7]. The major components of Uncaria are alkaloids, Rhy, and isorhynchophylline (IsoRhy) [8], and these are known to show biological effects such as vasodilation [9], hypertension, anti-platelet aggregation [10], and protective effects against neuronal damage [11]. In the Gou-teng plant, Rhy attributes for 28–50% and IsoRhy for 14% and apart from the above major alkaloids, trace levels of akuammigine, hirsuteine, hirsutine, corynantheine, dihydrocorynantheine, isocorynoxeine, and geissoschizine methyl ether were also seen [12,13]. Rhy exhibited several pharmacological effects such as anti-rhythmic, anti-hypertensive, anti-addictive, anticonvulsant, sedative, anti-anxiety, and neuroprotective activities in different experimental models [14,15,16,17] (Figure 1).

Inflammation is defined as the body’s immune response against harmful stimuli such as injury, toxic compounds, and pathogens. Cells mediate the healing process soon after injurious stimuli are removed [18,19]. Inflammatory cells mediate responses by cellular and molecular events such as redness, swelling, heat, pain, and loss of tissue function [20]. In acute inflammation, cellular and molecular events minimize injury or inflammation, whereas chronic inflammation predisposes the body to several inflammatory disease conditions such as rheumatoid arthritis, cardiovascular disease, asthma, etc. [21]. Due to sustained inflammatory response, monocytes and leukocytes are recruited to the inflammatory site followed by tissue injury. Microcirculatory events such as a change in vascular permeability, leukocyte recruitment, accumulation, and release of inflammatory mediators occur with mediators such as bradykinin and histamine [22,23].

Anti-inflammatory drugs encounter with the pathophysiology of inflammation and curb the tissue damage injury. Glucocorticoids and non-steroidal anti-inflammatory drugs (NSAID) are the two major types of anti-inflammatory drugs, and in fact, NSAID is the most widely used drug worldwide [24] and used to treat pain as well as acute and chronic inflammatory diseases. NSAID mediate the action by inhibiting COX involved in the production of prostaglandins and thromboxanes [25,26,27,28]. Despite the activity, they possess adverse effects on the human system such as gastrointestinal toxicity (perforation, dyspepsia, gastroduodenal ulcers, and gastrointestinal bleeding), nephrotoxicity (electrolyte imbalance, reduce glomerular filtration rate, nephrotic syndrome and chronic kidney diseases), and cardiovascular effects (hypertension, myocardial infarction, stroke, congestive heart failure, and other thrombotic events) [29]. NSAID can be of two classes, one that suppresses both cyclooxygenase-1 (COX-1) and cyclooxygenase-2 (COX-2) enzymes, such as ibuprofen, aspirin, and naproxen, and another class that selectively inhibits COX-2 (Coxibs), by targeting only the COX-2 pathway such as NS398, DuP697, celecoxib, valdicoxib, and rofecoxib [30]. The aftermath associated with NSAIDs are gastrointestinal discomfort, peptic ulcer disease, and gastrointestinal bleeding due to reduction in prostaglandins required for normal physiology [31].

Plants have been used as therapeutic agents in Ayurveda and traditional Chinese medicine. They act as reservoirs of secondary metabolites that can drastically reduce inflammation with fewer side effects. Secondary metabolites are synthesized from the mevalonate pathway, acetate pathway, and deoxyxylulose phosphate pathway (DXP) [32]. Alkaloids are secondary metabolites and are a class of amino acid-derived nitrogen compounds. They are classified as indoles, consisting of isoquinoline based on their carbon skeleton and pyridine-like alkaloids and ornithine, tyrosine, lysine, and tryptophan originated alkaloids concerning their biochemical precursors [33,34]. Plant-derived alkaloids are known for their effectiveness in treating inflammatory diseases [24,35]. Certain plants are rich in alkaloids, and their extracts are used as therapeutics to treat various ailments. Morphine was the first alkaloid to be discovered. Berberine is an isoquinoline alkaloid with anti-inflammatory, cardioprotective, and neuroprotective effects [36,37]. It has been shown to reduce endothelial inflammation, improve vascular health, lower lipid, and improve insulin resistance [38]. Caffeine has anti-inflammatory activity and has a beneficial effect on Alzheimer’s disease [39]. Other alkaloids with anti-inflammatory effects are galantamine [40], huperzine A, Rhy and IsoRhy, harmine, nicotine, etc. [41].

This review paper highlighted the current progress in the pharmacological effects of Rhy and its prospects as a preventive therapy for cardiovascular diseases.

## 2. Rhynchophylline and Nervous Disorders

Traditional Chinese medicine has been used in treating central nervous system (CNS) disorders. Neuroprotection can be defined as approaches that protect the CNS against injuries, neuronal infections, tumors, autoimmune disorders, and neurodegeneration that can lead to CNS disorders. Rhy has been found to be effective against CNS disorders such as drug addiction, epileptic seizures, vascular dementia, and cerebral ischemia [42]. It mediates neuroprotection by suppressing calcium channel suppression, opening potassium channels, and modulating neurotransmitters and metabolism [43].

Epilepsy is a chronic brain disorder delineated by a prolonged predisposition to generate seizures [2]. Encephalitis can induce inflammation with heightened levels of pro-inflammatory cytokines leading to epileptogenesis [44]. Activated nuclear factor kappa B (NFkB) can generate proinflammatory cytokines and reactive oxygen species leading to inflammation and neuronal apoptosis [45]. Mitogen-activated protein kinase (MAPK) is activated by N-methyl-D-aspartate (NMDA) and non-NMDA/kainate receptors of glutamate and play a pivotal role in the development of epilepsy [46]. Rhy is used in the treatment of epilepsy, which is a chronic nervous system disorder with unusual seizures and sensations [47]. The anticonvulsant effect of *U. rhynchophylla* was reported in kainic acid (KA)-induced epileptic seizures [48]. Rhy can act as a noncompetitive NMDA glutamate receptor antagonist [49] and inhibit calcium influx and prevent glutamate-induced neuronal death in vitro [50]. It was also found that both the *U. rhynchophylla* plant and Rhy reduced KA-induced epileptic seizures by attenuating c-Jun amino terminal kinase phosphorylation (JNKp) expression and NFkB activation [51]. Using network pharmacology, around 20 genes were identified as targets for Rhy in treating epilepsy. Rhy could exert the pharmacological effects by interacting with multiple genes that share a protein domain that can be explored in the evolution of a safe and multi-targeted effectual compound for anti-epilepsy therapy [52].

Rhy has been proved to have anti-addictive effects [53] and is used to treat symptoms related to drug addiction. Methamphetamine (MA) is a widely abused psychostimulant that can lead to several neurodegenerative alterations in the human brain [2]. It causes neuronal damage via ionotropic glutamate receptors and induces Ca^2+^ influx that can lead to mitochondrial dysfunction and reactive oxygen species (ROS) generation [54]. Rhy has been ascertained to be a calcium channel antagonist in blood vessels and acts as a non-competitive antagonist for glutamate receptors [55]. MA-induced Ca^2+^ influx was decreased by Rhy in rat neurons in vitro through calcium channel blockage/through inhibition of ionotropic glutamate receptor [56]. Rhy showed its therapeutic effects in MA-induced conditioned place preference (CPP) rats by decreasing p-Fos and phosphorylated cAMP response element-binding protein (p-CREB) levels in the brain [57]. The anti-addictive effects of Rhy were also studied in ketamine-dependent rat models that indicated a significant decrease in the levels of nuclear receptor-related-1 (Nurr1), cAMP response element-binding protein (p-CREB), and brain-derived nucleophilic factor (BDNF) in the hippocampus associated with ketamine addiction [15]. Rhy also effectively repressed the effect of amphetamine-induced conditioned place preference by reducing the levels of central neurotransmitters in the rat brain [58] as well as reduced NR2B and protein expression in the hippocampal CA1 areas and medial prefrontal cortex in rats [59].

Alzheimer’s disease (AD) is a demyelinating and degenerative disease that widely affects the health of elderly people and is also the most common cause of dementia [60]. The disease pathogenesis is characterized by senile plaques or amyloid beta (Aβ) plaques, neurofibrillary tangles encompassing hyperphosphorylated aggregated tau protein, chronic neuroinflammation associated with stimulated microglia and astrocytes, and neurodegeneration [61,62,63]. Terpene indole alkaloids present in Uncaria plants can inhibit and destabilize amyloid-beta protein, a hallmark of AD [64]. Rhy and its isomer isorhychophyline have been found to exert neuroprotective effects by rescuing PC12 neuronal cells from death after the Aβ challenge [65,66]. It was found to mediate the protective effect through reducing Ca^2+^ overload as well as tau protein hyperphosphorylation [67]. Rhy rescued hippocampal synaptic dysfunction in AD and acts as an inhibitor of ephrin type A-receptor 4-precursor (EphA4) tyrosine kinase [68]. Oral administration of Rhy reduced EphA4 activity in APP/PS1 transgenic mice as well as restored impaired recurrent potentiation in the hippocampus, substantiating the beneficial effects of Rhy on synaptic dysfunction in AD [69]. Although Rhy is very effective in reducing the pathogenesis of AD, it has certain limitations such as reduced solubility and poor bioavailability in brain tissue, and low permeability across the blood–brain barrier [70,71]. The efficiency was improved using nanotechnology. mPEG-PLGA nanoparticles coated in Tween 80 (T80-NPS-RIN) were loaded with Rhy to improve treatment efficacy and a high fold increase in bioavailability and bioaccumulation was observed in brain tissues [72]. A combinatorial effect of both Rhy and gastrodin (a glucoside isolated from the Chinese herb *Gastroida elata*) was shown to be effective in ischemia-induced brain damage. Gastrodin is known for its various pharmacological effects [73]. Both the components suppressed inflammasome activation, and gastrodin upregulated miR-21-5p target thioredoxin interacting protein (TXNIP) while Rhy upregulated miR-331-5p target TRAF6 in mice [74].

The anti-migraine effect of Rhy was provided by Lai et al. in nitroglycerin-induced migraine rat models. The study proved the protective effects of Rhy by inhibiting MAPK/NF-kB pathway against oxidative stress [75]. The role of NF-kB in the advancement of migraine through initiating oxidative stress was previously studied (84). However, Rhy also attenuated LPS-induced inflammation in microglial cells via MAPK/NF-kB pathway [76]. Early Brain Injury (EBI) after subarachnoid hemorrhage (SAH) was effectively regulated by Rhy through reducing the concentrations of ROS and malondialdehyde (MDA) in the hippocampus [77].

## 3. Rhynchophylline in Atherosclerosis and Other Cardiovascular Diseases

Atherosclerosis is an important cardiovascular disease that affects both the heart and brain, manifested by fatty streaks and plaques in blood vessels [78]. It is currently considered a chronic inflammatory condition with several risk factors, such as hypercholesterolemia, hypertension, diabetes, age, gender, atherogenic diet, increased lipoprotein A [79], obesity, family history, psychiatric factors, and increased blood clotting [80], that are referred to as coronary artery disease (CAD).

Major inflammatory markers of atherosclerosis are high sensitivity C-reactive protein (hs-CRP), TNF*α,* and IL-6 [81].

Lifestyle diseases such as type-2 diabetes mellitus (DM), obesity, and metabolic syndrome are known non-infectious pandemics and are risk factors in the development of atherosclerotic cardiovascular diseases (ASCVDs). They stimulate plaque formation via modulating pathways associated with cardiovascular risk [82]. The factors dyslipidemia, hyperglycemia, and insulin resistance can mediate physiological changes leading to the development of atherogenic LDL, advanced glycation end products (AGE), and activates proinflammatory signaling cascade instigating atherosclerotic lesions.

Patients with type 2 diabetes-associated coronary artery disease express higher levels of cytokines and mediators such as TNF-α, interleukins, MCP-1, cyclophilin A, CD-36, and LOX-1 [83] (Table 1).

Progression of atherosclerosis associated with diabetes can be prevented by specifically targeting inflammatory pathways. Therapies involving small molecule anti-inflammatory drugs such as salicylates [84], inflammatory inhibitors such as TNF-α inhibitors, and IL-1β antagonists [85] reduce cardiovascular risk in diabetic patients. Metformin, a first-line anti-diabetic drug used for the treatment of diabetes, has also been proved to be effective in alleviating ASCVD [86]. Complementary therapy based on monoclonal antibodies to PCKSK9 (proprotein convertase subtilisin-kexin type 9), the SGLT-2 (sodium-glucose cotransporter-2) inhibitors, GLP-1 RAs (glucagon-like peptide-1 receptor agonists), and novel therapy based on RNA therapeutics and targeting the RAGE (receptor for advanced glycation end-products) can provide cardioprotection in diabetes [87]. All these therapies are potentially effective in reducing inflammatory mediators.

**Figure 2:**
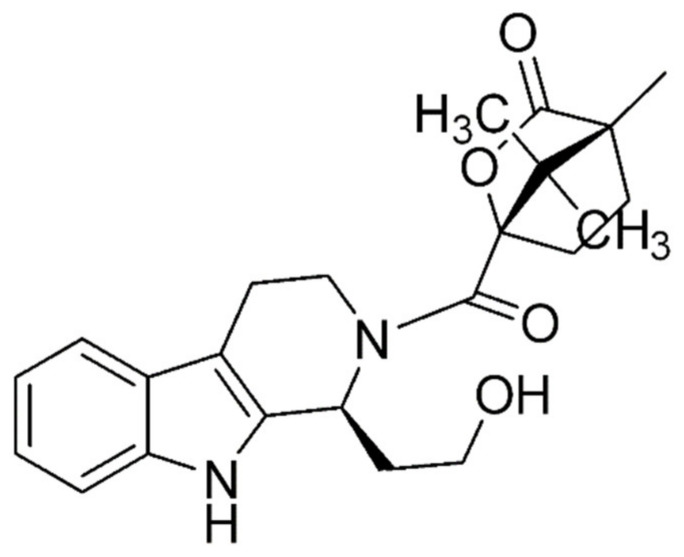
Chemical structure of novel rhynchophylline analog Y396.

In rats, Rhy analog G2 was shown to ameliorate diabetes-induced endothelial dysfunction in mesenteric arteries. It also upregulated eNOS expression and contributed to vascular relaxation [88]. A novel Rhy analog, Y396 (Figure 2), inhibited the tyrosine kinase activity of EGFR as well as restored endothelial vascular relaxation without affecting vascular structure by downregulating Nox2 and Nox4 [89]. EGFR is activated in diabetes and atherosclerosis. Y396 mitigated diabetes by decreasing oxidative stress in Type-1 diabetes mellitus mice and primary mice aorta endothelial cells. Apart from the role of Rhy in hypertension and septic shock, it was found to have anticoagulation and anti-endotoxemic effects [16,90]. Antiarrhythmic effects were also displayed by declining myocardial excitability and prolonging the refractory period in guinea pigs [91]. Rhy significantly inhibited platelet aggregation and reduced the levels of thromboxane A2 (TXA2) and thromboxane B2 (TXB2) in collagen-induced groups but did not alter in the acetic acid-induced group [92]. It is evident that Rhy prevented thrombosis and platelet aggregation, thereby conferring anticoagulant activity which is required for preventing myocardial infarction.

The initial stages of atherosclerosis involve plaque formation followed by inflammation, endothelial dysfunction, and the interaction between platelet and endothelium. As macrophages have a low affinity towards non-oxidized LDL, reducing the levels of LDL can prevent its further oxidation. However, there arises a need for persuasive preventive medicine that can substantially lower LDL glycation or oxidation as well as mediate lesion formation regression. Nanotechnology unveils new avenues for enhanced treatment, especially in cardiovascular diseases. It imparts the potential of targeted drug delivery with fewer side effects. As with Alzheimer’s disease treatment, intravenous administration of Rhy-loaded nanoparticles may be used to enhance the treatment efficacy in early atherosclerosis thereby reducing macrophage foam cell formation in arteries. Nanoparticles can be internalized by macrophages and Rhy may either act by blocking the uptake of oxLDL by macrophages or downregulating the receptor mediating the uptake.

CVD is correlated with several predisposing disorders such as hypocholesteremia [93], hypertension, and diabetes. Both diabetes and hypertension are stimulated by certain common factors that promote atherosclerosis, vascular inflammation, endothelial dysfunction, and structural remodeling leading to microvascular and macrovascular diseases [94]. In traditional Chinese medicine, *Uncaria* spp. are often used to cure cardiovascular disorders such as bradycardia, arrhythmia, and hypertension [42]. *U. rhynchophylla* extracts were used in the treatment of hypertension as Rhy displayed cardioprotective effects through calcium transport dysfunction that comprises majorly extracellular calcium influx and release of intracellular calcium by occluding voltage-gated calcium channel and receptor regulation of calcium channel [95].

Anti-hypertensive activities of Rhy have been reported in many animal models [42] and are attributed to its vasodilatory effect by decreasing calcium sensitivity in smooth muscles or through inhibition of L-type calcium channels [96,97]. Hypertension is a cardiovascular syndrome that causes functional and structural changes in the heart and blood vessels. The etiology includes vascular injury and vascular dysfunction leading to target organ injury [98]. Endothelial progenitor cells (EPCs) are majorly involved in vascular repair and in improving tissue ischemic; however, obesity and cardiovascular diseases can impend the function, senescence, and trans-differentiation of EPCs [99,100]. Previously, Rhy was reported to inhibit vascular smooth muscle cell proliferation and reduce Ang II-induced cardiomyocyte hypertrophy [101,102]. Recently, Rhy was found to decelerate Ang II-induced EPC senescence as well as enhanced autophagy and protected EPC against Ang II via the AMP-activated protein kinase (AMPK) signaling pathway [103]. In spontaneous hypertensive rats (SHR), Rhy was shown to upregulate phosphorylation of both Akt and Src. Moreover, Rhy protected endothelial function and improved endothelial-dependent relaxation in renal arteries from SHR via Src-PI3K/Akt-eNOS signaling cascade [104]. Furthermore, Rhy rescued the oxidized lipoprotein-induced autophagy in human umbilical vein endothelial cells [105]. Rhy regulated the expression of Bax, Bcl-2, c-Myc, c-Fos, and TGF-*β*_1_ as well as promoted vascular adventitial fibroblasts apoptosis in the thoracic aorta of SHR. Subsequently, it accelerated thoracic aorta wall reconstruction and attenuated the deposition of the extracellular matrix [106].

Septic shock can lead to myocardial dysfunction and no therapies are targeting myocardial depression. Thus, there arises a call for the identification of novel therapies that could improve cardiac dysfunction as well as increase survival in sepsis. Rhy possesses anti-inflammatory, antiarrhythmic, anti-hypertensive, and neuroprotective effects [107]. Rhy possesses chemical similarity with that of yohimbine [108] which was previously reported to prevent myocardial dysfunction [109]. It was implicated that Rhy may also possess similar activity was proved to be effective in reducing cardiac dysfunction and improving the survival in LPS challenged mice. Rhy intervened the effect via suppression of IκBα phosphorylation and inhibition of myocardial TNF-α and IL-1β in unfiltered macrophages during endotoxemia [15]. Qin et al. have reported that Rhy modulated Ca^2+^ and mitochondrial membrane potential (MMP) levels in cardiomyocytes. There was a significant increase in cell viability and attenuated apoptosis in myocardial ischemia-reperfusion (MI/R)-induced cardiomyocytes [110].

Moreover, Rhy has been found to be less toxic to cells. Rhyncophylline substantially reduced the LPS lethality of mice and increased the survival rate of mice pretreated with Rhy 16 mg/kg after the LPS challenge (43% compared to the LPS alone (9.5%) [16]. Rhy, at concentrations of 200 µM for 48 h, did not exhibit significant toxicity in neuronal cultures compared with the untreated cultures [56]. Rhy was also nontoxic to PC12 cells up to the dose of 1.0 mmol/L [17]. All these are suggestive of the nontoxicity of Rhy which can be better utilized as a safety component in preventive medicine.

**Figure 3:**
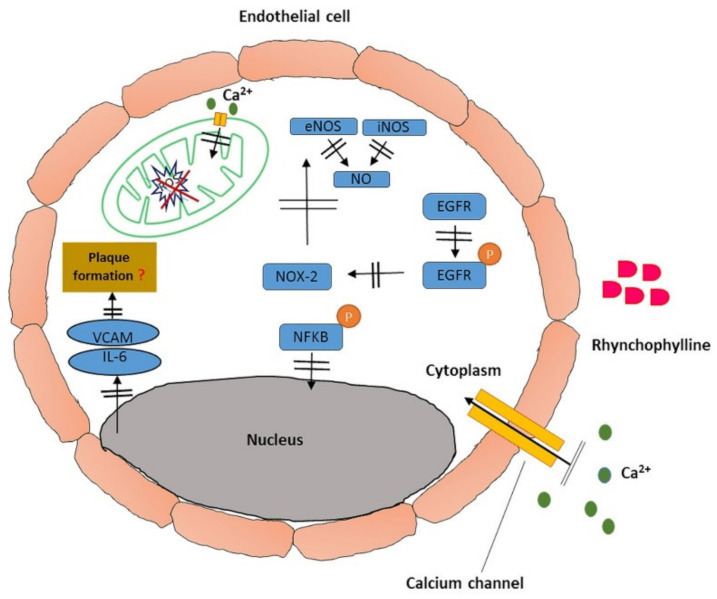
Diagram of the effects of Rhy in preventing plaque formation in endothelium: Rhymodulates calcium channels and mitochondrial membrane potential to inhibit the expression of pro-inflammatory cytokines and enzymes. It prevents tyrosine kinase activity of epidermal growth factor receptors to suppress oxidative stress.

Although there are several reports on the prospective of Rhy in the reconstruction of aorta wall, modulation of Ca^2+^ and MMP levels in cardiomyocytes, against myocardial infarction, suggestive of repair and protection in later events in advanced atherosclerosis, little is known about its impact on early atherosclerosis, especially in diabetes. Preventing early atherosclerosis can block further complications associated with it and improve the lifestyle of individuals especially in diabetic patients. Consequently, there arises an upsurge for exploring the role of Rhy in reducing the levels of oxLDL, modulating macrophages, or regressing plaque formation. This area remains unexplored, and it may contribute to the progressive development of atherosclerotic preventive medicine (Figure 3).

## 4. Pharmacological Effects of Rhynchophylline in Other Diseases

It was reported that Rhy significantly reduced LPS-induced inflammatory mediators such as NO, TNF-α, and IL-1β via suppressing NF-kB, ERK, and p38 MAPKs in N9 microglial cells. Furthermore, Rhy suppressed nerve cell excitability and relaxing blood vessels [107]. In LPS-challenged mice, Rhy reduced TNF-α immunoreactivity in cardiac infiltrated macrophages and inhibited the expression of pro-inflammatory cytokines via MAPK, c-Jun N terminal kinase (JNK), and NF-kB signaling pathways [57,111]. Rhy exhibited anti-inflammatory and anti-apoptotic effects by triggering the nuclear factor E2-related factor 2-antioxidant response element (Nrf2/ARE) pathway [78].

Asthma is a chronic inflammation characterized by inflammation of the airway as well as remodeling and hyper-responsiveness with an increase in cytokine levels and the patients experience refractory asthma. Although anti-inflammatory therapies comprising bronchodilators and corticosteroids are effective in controlling acute events as well as improving the inflammatory symptoms, they do not alleviate airway remodeling. This urges the need for an efficient asthmatic therapy that could reverse the remodeling process [112,113]. *Uncaria rhynchophylla* was found to attenuate the effects of asthma [114] and Rhy effectively alleviated asthmatic inflammation by suppressing JAK2/STAT3 signaling pathway thereby reducing oxidative stress and suppressing autophagy-related proteins [115]. Allergic asthma was elicited by ovalbumin and hyperplasia of airway smooth muscles were induced by transforming growth factor-β1 (TGF-β1). Rhy mitigated inflammation both in vivo and in vitro by blocking Smad and MAPK signal transduction pathways [116].

Listeriosis is caused by *Listeria monocytogenes* and *U. rhynchophylla* herb has been used in TCM in treating Listeriosis. Listeriolysin O (LLO) can activate rat intestinal microvascular endothelial cells (RIMECs) to establish RIMEC injury models. Both Rhy and IsoRhy increased the cell viability and upregulated NO levels but inhibited endothelin-1(ET-1) release [117].

The expression levels of bone marrow mesenchymal stromal cells (BM-MSCs) proliferation and differentiation-related transcription genes are altered by Rhy. The BM-hMSCs metabolism was shifted to aerobic glycolysis on contrary to mitochondrial oxidative phosphorylation. This activity of Rhy-induced BM-hMSCs modification augmented the stem cells metabolic activity which can be established to enhance cell transplantation methods [118]. The highlights of pharmacological activities of Rhy have been summarized in Table 2.

## 5. Conclusions

Uncaria species have been widely used in traditional Chinese medicine alleviating several neurological disorders as well as cardiovascular diseases. The active component Rhy exhibited a similar effect to that of the plant. This review especially highlighted the recent studies of Rhy in both in vitro and in vivo models. Rhy exhibited protective effects against Alzheimer’s disease, epilepsy, drug addiction, migraine, diabetes, and hypertension mediated via anti-inflammatory and antioxidant activities. Recent advancements of Rhy loaded nanoparticles could upsurge the treatment efficacy in Alzheimer’s disease by crossing the blood–brain barrier. Despite the studies in neuro disorders, there exist lacunae in the effects of Rhy in reducing early atherosclerosis associated with lifestyle diseases mostly patients with diabetes mellitus. Diabetes mellitus hastens and mediates the formation of atherogenic LDL and predisposes to atherosclerosis. It is a requisite to understand and explore the anti-atherosclerotic effects of Rhy thereby assuaging and averting plaque formation, especially in diabetes. Moreover, nanotechnology using Rhy-loaded nanoparticles can be used as a preventive intervention in alleviating early atherosclerosis and could also be used as adjunctive therapy to prevent cell damage and myocardial infarction. However, future studies should investigate and explore the diverse activities of Rhy in cardioprotection.

## Figures and Tables

**Table 1:** Markers expressed by activated macrophages and endothelial cells during early and late stages of plaque formation.

Markers Expressed by Activated Macrophages/Endothelial Cells in Early Stage of Plaque Formation	Markers Expressed by Activated Macrophages/Endothelial Cells in Later Stage of Plaque Formation
E-selectin	VCAM-1
E-Cadherin	Fibrinogen
P-selectin	MCP-1
MCP-1	MPO
MMP	CD14
VCAM-1	CD16
LOX	CCR2
CD36	
Inflammatory markers	Inflammatory markers
Cyp A	CRP
hs-CRP	TNF-α
TNF-α	IL-18
IL-1	
IL-6	

**Table 2:** Summary of pharmacological activities of Rhy.

Diseases	Study Highlights of Rhy	References
Epilepsy	Noncompetitive NMDA glutamate receptor antagonist	Hsieh et al., 2009 [49]
	Inhibited calcium influx and prevented glutamate-induced neuronal death in vitro	Kang et al., 2002 [50]
	Attenuated JNKp expression and NFkB activation	Shimada et al., 1999 [51]
Addiction	Blocked calcium channel through inhibition of ionotropic glutamate receptor	Xu et al., 2012 [56]
	Decreased the levels of p Fos, Nurr, p-CREB, and BDNF in the hippocampus	Liu et al., 2014 [57] Yung et al., 2018 [15]
Alzheimer’s	Reduced Ca^2+^ overload and tau protein hyperphosphorylation	Xian et al., 2012 [67]
	Inhibitor of ephrin type A-receptor 4-precursor (EphA4) tyrosine kinase	Fu et al., 2014 [68]
	Nanoparticle T80-NPS-RIN improved bioavailability and bioaccumulation	Xu et al., 2020 [72]
Migraine	Inhibited MAPK/NF-kB pathway against oxidative stress	Lai et al., 2019 [75]
	Reduced the concentrations of ROS and malondialdehyde (MDA) in the hippocampus	Song et al., 2012 [77]
Diabetes	Rhy analog G2 ameliorated diabetes-induced endothelial dysfunction in mesenteric arteries and upregulated eNOS expression	Guo et al., 2017 [88]
	Novel Rhy analog, Y396 inhibited the tyrosine kinase activity of EGFR and down regulated Nox2 and Nox4	Wang et al., 2020 [89]
Hypertension	Vasodilatory effect by decreasing calcium sensitivity in smooth muscles or through inhibition of L-type calcium channels	Li et al., 2013 [96] Hao et al., 2014 [97]
	Inhibited vascular smooth muscle cell proliferation and reduced Ang II-induced cardiomyocyte hypertrophy	Zhang et al., 2008 [101] He et al., 2010 [102]
	Improved endothelial-dependent relaxation in renal arteries from spontaneous hypertensive rats via Src-PI3K/Akt-eNOS signaling cascade	Hao et al., 2017 [104]
Septic shock	Suppressed IκBα phosphorylation, inhibited myocardial TNF-α and IL-1β in unfiltered macrophages during endotoxemia	Yung et al., 2018 [15]
	Increased cell viability and attenuated apoptosis in myocardial ischemia-reperfusion (MI/R)-induced cardiomyocytes	Qin et al., 2019 [110]
Asthma	Reduced asthmatic inflammation by suppressing JAK2/STAT3 signaling pathway thereby reducing oxidativestress and suppressing autophagy-related proteins	Li et al., 2021 [115]
	Blocked Smad and MAPK signal transduction pathways	Wang et al., 2019 [116]
Listeriosis	Increased the cell viability of intestinal microvascular endothelial cells and upregulated NO levels but inhibited endothelin-1(ET-1) release	Chen et al., 2010 [117]

## Data Availability

Not applicable.

## Abbreviations

Rhy	Rhynchophylline
IsoRhy	Isorhynchophylline
oxLDL	Oxidized low-density lipoprotein
EPC	Endothelial progenitor cell
NSAID	Non-steroidal anti-inflammatory drugs
COX	Cyclooxygenase
IL-1	Interleukin-1
IL-8	Interleukin-8
TNF-α	Tumor necrosis factor α
IFN-γ	Interferon γ
hsCRP	High-Sensitivity C-reactive protein
ROS	Reactive oxygen species
JNKp	c-Jun amino terminal kinase phosphorylation
MAPK	Mitogen activated protein kinase
Cyp A	Cyclophilin A
MMP	Mitochondrial membrane potential
MMP	Matrix metalloproteinases
Nurr1	Nuclear receptor-related-1
p-CREB	cAMP response element-binding protein
MCP-1	Monocyte chemoattractant protein-1
ICAM-1	Intracellular adhesion molecule-1
VCAM-1	Vascular cell adhesion molecule-1
NF-kB	Nuclear factor kappa-B
LOX-1	Lectin like ox-LDL receptor-1
SR-A1	Scavenger receptor-1
CD36	Cluster of differentiation 36
LPS	Lipopolysaccharide
CNS	Central nervous system
EphA4	Ephrin type A-receptor 4-precursor
NMDA	N-methyl-D-aspartate
BDNF	Brain-derived nucleophilic factor
JNKp	c-Jun amino terminal kinase phosphorylation
CPP	Conditioned Place Preference
EBI	Early Brain Injury
TXA2	Thromboxane A2
TXB2	Thromboxane B2
AMPK	AMP-activated protein kinase
SHR	Spontaneous hypertensive rats
GLP-1 RAs	Glucagon-like peptide-1 receptor agonists
SGLT-2	sodium-glucose cotransporter-2
AGE	Advanced glycation end products
PCKSK9	Proprotein convertase subtilisin-kexin type 9
RAGE	Receptor for advanced glycation end-products
DM	Diabetes mellitus
ASCVD	atherosclerotic cardiovascular diseases
Ang	Angiotensin
MA	Methamphetamine
KA	Kainic acid
EPC	Endothelial progenitor cell
EGFR	Endothelial growth factor receptor
